# Catheter Ablation for Atrial Fibrillation in Patients with Heart Failure

**DOI:** 10.5935/abc.20180066

**Published:** 2018-04

**Authors:** Mauricio Scanavacca, Edimar Alcides Bocchi

**Affiliations:** 1 Instituto do Coração (InCor), Hospital das Clínicas da Faculdade de Medicina da Universidade de São Paulo, São Paulo, SP - Brazil

**Keywords:** Heart Failure, Atrial Fibrillation, Catheter Ablation / trends, Atrial Remodeling, Amiodarone

In the last years, atrial fibrillation (AF) and heart failure (HF) have been the two major epidemic syndromes in cardiology and they frequently coexist.^1^ HF increases mean right and left atria pressures promoting their progressive dilation. Such mechanical electro-anatomic remodeling predisposes to atrial fibrosis and electrical heterogeneity, increases ectopic rhythm formation and ultimately induces AF.^2^

A new AF episode, in turn, immediately induces loss of atrial contraction, increases mean heart rate and provokes an important irregularity on ventricular contractions decreasing the heart’s pump function performance. Therefore, around 50% of patients who present with new-onset congestive HF have atrial fibrillation and up to one-third of patients with new-onset AF have congestive heart failure.^2^

The Framingham study demonstrated that in AF patients, occurrence of HF was associated with significant increase in mortality, as well as in HF patients, a new AF development was associated with significant rise on mortality.^3^ Therefore, there is a biological rationale for the prevention and treatment of AF associated with HF. The targets would be ventricular control, especially rhythm control.

Several pharmacological studies have failed to demonstrate clinical benefits in maintaining sinus rhythm compared to rate control in patients with normal or abnormal left ventricle function.^4-6^ In the AFFIRM trial, the management of atrial fibrillation with rhythm-control strategy offered no survival advantage over the rate-control strategy, and patients had higher rate of hospitalization. The potential explanation for that was the antiarrhythmic drugs’ adverse effects.^4^ In patients with left ventricle dysfunction, the use of antiarrhythmic drugs safely recommended for this condition, such as dofetilide and amiodarone, also did not show any hard endpoint benefits.^5,6^

Catheter ablation for AF has emerged as the most effective strategy to maintain the sinus rhythm in patients with paroxysmal and persistent AF and has been used worldwide.^7,8^ However, there is a paucity of studies investigating hard endpoints as mortality reduction in patients with HF with catheter ablation. The study “A randomized controlled trial of catheter ablation versus medical treatment of atrial fibrillation in heart failure (the CAMTAF trial)“ was able to demonstrate an improvement in left ventricular ejection fraction (LVEF) with ablation in patients with persistent AF.^9^ Additional advantages were observed in the *“Ablation versus Amiodarone for Treatment of Atrial Fibrillation in Patients with Congestive Heart Failure and an Implanted ICD (The AATAC) trial”.* Di Biase et al^10^ showed that ablation was superior to amiodarone in maintaining sinus rhythm, improving LVEF, improving survival rates and decreasing hospitalization for HF.

More recently, an additional enthusiasm comes up with the report of *“Catheter ablation for atrial fibrillation with heart failure (Castle-AF) trial”.* Marrouche et al confirmed observations of the AATAC trial, showing that catheter ablation of AF significantly reduces mortality in patients with HF, as compared with medical therapy.^11^

*CASTLE-AF* is a multicenter study, conducted from January 2008 through January 2016, and involving a total 33 sites in Europe, Australia, and the United States. In this study, 263 patients with symptomatic paroxysmal or persistent AF were randomly assigned to undergo AF catheter ablation (179) or medical treatment (184), using rate or rhythm control strategies. All the patients had New York Heart Association (NYHA) class II, III, or IV HF, a LVEF of 35% or less, and an implanted defibrillator. The primary end point was notably hard, a composite of death from any cause and hospitalization for worsening HF. The final results were obtained after a median follow-up of 37.8 months and favored catheter ablation comparing to medical therapy. In the ablation group, 63% of patients were in sinus rhythm at 60 months versus 22% in the medical-therapy group. The primary composite end point occurred in 51 (28.5%) patients in the ablation group and in 82 (44.6%) patients in the medical therapy group (HR = 0.62; P = 0.007).

There was a significant reduction of all-cause mortality in the ablation group (13.4% vs. 25.0%), HR = 0.53, P = 0.01 and from cardiovascular causes (11.2% vs. 22.3%); HR = 0.49; P = 0.009. Additionally, patients undergoing catheter ablation showed reduced hospitalization rate in consequence of worsening heart failure (20.7%) comparing to medical treatment (35.9%), HR = 0.56, P = 0.004. Furthermore, catheter ablation reduced the burden of AF, increased the distance walked in 6 minutes, and improved the LVEF (8%). An important detail from this study was the observation that the mortality benefit of ablation emerged just after 3 years of follow-up.

These observations are unique since it is the first trial on catheter ablation field designed to show both, superiority in maintaining the sinus rhythm and mortality reduction comparing to medical therapy. However, CASTLE-AF trial has some important limitations as highly patient selection - from 3,013 patients assessed for eligibility, just 263 were finally included in the primary analysis. Investigators were not blinded treatment randomization, and a number of patients crossed over to the other treatment group. Additionally, the procedures were performed in high-volume medical centers with very experienced operators. Also, inclusion criteria of patients to the CASTLE-AF trial included absence of response to (45-47%), unacceptable side effects from (12-14%), and unwillingness to take antiarrhythmic drugs (40-43%). In fact, in the CASTLE-AF study the AF ablation was not tested in patients under acceptable rate control or rhythm control. So, new studies are needed to confirm such important observations.

Benefits of catheter ablation of AF have also been suggested in a recent retrospective study evaluating HF patients with preserved ejection fraction HFpEF.^12^ Two hundred-thirty AF patients with HF, 133 HFpEF and 97 patients with reduced ejection fraction (HFrEF) underwent catheter ablation. After a mean follow-up of 12 months, postablation outcomes as in-hospital adverse events, symptoms according to the Mayo AF Symptom Inventory (MAFSI), NYHA functional class, and freedom from atrial arrhythmia] were recorded. Ablation procedure (pulmonary vein isolation, pulmonary vein isolation with roof line, complex fractionated atrial electrograms), procedural time, fluoroscopy duration, and radiofrequency time were comparable between these groups.

After ablation, the incidence of acute HF across these groups was similar. Both groups improved in MAFSI and NYHA functional class. Before ablation most of the patients were in NYHA functional class II, but after ablation the majority of patients shifted to class I from the more advanced classes. Preablation LVEF showed no correlation with freedom from atrial arrhythmia or repeat ablation rate. These results remained the same even after stratification based on AF phenotype. At 12 months postablation, all-cause hospitalization and cardiovascular hospitalization were similar for these patients. Also, previous study on AF ablation in HFpEF has suggested that AF can be effectively and safely treated with a composite of repeat procedures and pharmaceuticals. However, larger randomized controlled studies are also needed to confirm the benefits of AF ablation in HFpEF.^13^

In conclusion, HF and AF are widely distributed diseases and difficult-to-treat conditions due to their synergistic effect. Once installed, a vicious circle is established, which significantly worsens the patient’s prognosis. No mortality or hard endpoint benefits have been demonstrated with the most commonly used antiarrhythmic drugs. Evidence has been generated in the last decade in favor of AF ablation in selected patients with AF with preserved or reduced LVEF.

Based on these new data, catheter ablation has already been considered as first-line therapy in patients with paroxysmal or persistent AF and HF.^14^ Evident benefit can be obtained in patients in which AF is the main cause for HF (tachycardiomyopathy).^15^ However, we still need to develop new markers and tools to better define ideal ablation techniques and candidates, especially for patients under acceptable rhythm or rate control.
